# Discovery and development of steroidal enzyme inhibitors as anti-cancer drugs: state-of-the-art and future perspectives

**DOI:** 10.1080/14756366.2025.2483818

**Published:** 2025-04-02

**Authors:** Bruno Cerra, Antimo Gioiello

**Affiliations:** Department of Pharmaceutical Sciences, University of Perugia, Perugia, Italy

**Keywords:** Anticancer steroids, breast cancer, enzyme inhibitors, prostate cancer, steroidogenesis inhibitors

## Abstract

Steroidal compounds have emerged as effective therapeutic agents in oncology. Beyond natural-occurring and synthetic steroids that act as cytotoxic anti-tumoral agents, steroidal derivatives can be designed to mime the endogenous substrates of key metabolic enzymes in steroidogenesis, thus reducing the circulating levels of relevant oestrogenic and androgenic hormones responsible for cancer survival and proliferation. Therefore, enzyme inhibition represents an intriguing endocrine approach for the treatment of hormone-dependent tumours, such as breast and prostate cancer, with well-known approved drugs and several *pre*-clinical and clinical candidates under investigation. This review summarises the key advancements over the past decade (2014–2024) in the development of steroidal enzyme inhibitors endowed with anticancer activity, illustrating their mechanisms of action, therapeutic potential, drug design approaches, and current clinical applications. Furthermore, we discuss challenges related to drug resistance, off-target effects, and future strategies to optimise their efficacy in oncology.

## Steroids: an old scaffold for new anti-cancer drugs

The search for novel cancer therapeutics has placed a growing emphasis on the development of targeted therapies able not only to face the intricate and multifactorial molecular pathways at the basis of cancer development and progression^1–3^, but also to overcome drug-resistance and interindividual variability phenomena^4^^,^^5^. Within the small molecule-based armamentarium currently available^6^, the class of steroidal compounds occupies a first-rate position as chemotherapeutic agents^7–12^. Indeed, structurally diverse steroids have been developed as drugs^13–15^ or used as lead compounds/molecular probes within anticancer drug discovery campaigns to gain insight into specific cancer molecular mechanisms^10^^,^^12^. Steroidal compounds can act both as cytotoxic agents, through non-hormonal targets, and as anti-hormonal/anti-proliferative molecules by modulating the activity of key hormonal receptors or enzymes^16^^,^^17^. A plethora of natural-occurring and rationally-designed semi-synthetic steroids can act as cytotoxic agents against different human cancer cell lines through various specific or aspecific modes of action^18^. However, these compounds are generally recognised to be poorly selective and therefore represent the preferred therapeutic choice only at the advanced stage of disease and/or when resistance occurs during first-line therapy. On the other hand, hormone-dependent cancers, such as ER+/HER2-breast cancer, prostate cancers and castration-resistant prostate cancer, and some forms of endometrial, ovarian and adrenal cancers, can be effectively treated with hormone receptor modulators or steroidogenesis enzyme inhibitors^19^. Hormone-dependent cancers are the most common non-cutaneous cancers worldwide with almost 1 million patients and over 100,000 deaths only in the United States in 2024. In particular, breast and prostate cancer still represent the most commonly diagnosed cancer and the second leading cause of cancer death in developed countries among women and men, respectively^20–22^. However, the last half-century has witnessed a remarkable improvement in terms of 5-years survival outcomes (+21% for breast cancers and +40% for prostate cancer) thanks to faster and more efficient diagnosis, public awareness and screening campaigns, and, above all, the pharmacological manipulations of the endocrine mechanisms at the basis of these malignancies by means of steroidal receptors modulators and steroidogenesis enzyme inhibitors^23^^,^^24^.

Steroidal modulators of hormone receptors are a well-established endocrine cancer therapy in terms of efficacy and safety profile, with several blockbuster drugs on the market and just as many candidates under clinical development^25^^,^^26^. On the other hand, enzyme inhibitors are able to interfere with key steroidogenic biosynthetic pathways and to reduce the circulating levels of relevant oestrogenic and androgenic hormones involved in cancer cell survival and proliferation, thus representing a valid endocrine therapy for the treatment of hormone-dependent cancers^27^. Besides the direct effect in promoting the reduction of the level of endogenous steroid hormones, steroidal enzyme inhibitors also modulate innate and adaptive immunity and regulate the tolerogenesis, thus further counteracting the onset or progression of malignancy^28^. As a complement and update of previously reviewed articles detailing the clinical relevance of steroidal modulators of hormone receptors and anti-cancer cytotoxic steroids^10–12^^,^^16–18^^,^^25^^,^^26^, herein we have summarised the key advancements made over the past decade (2014–2024) in the discovery and development of anti-cancer steroids that act as enzyme inhibitors, shedding light on their mechanisms of action, rational medicinal chemistry design, and clinical applications. Furthermore, the challenges related to drug resistance and off-target effects, as well as the future strategies and directions in the field are critically discussed in the review.

## Steroidogenesis and its role in hormone-dependant cancers

**Figure 1.**
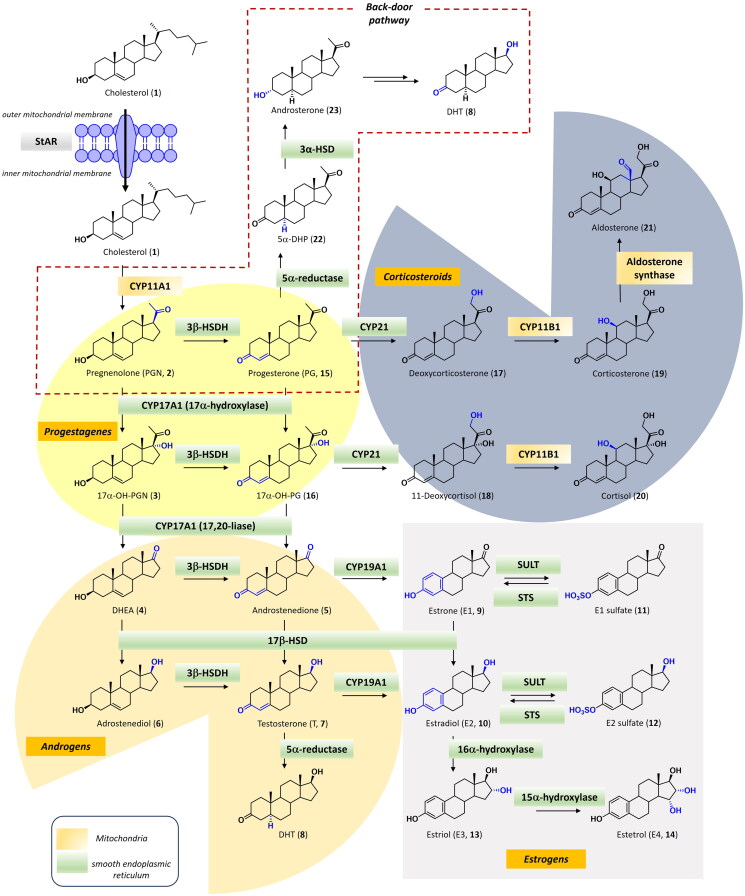
Steroidogenesis of androgens, oestrogens, progestogens, glucocorticoids and mineralocorticoids *via* classical and backdoor pathways. *Legend:* PGN (pregnenolone), PG (progesterone), T (testosterone), E1-4 (oestrogens), DHEA (dehydroepiandrosterone), DHT (dihydrotestosterone), 5α-DHP (5α-dihydroprogesterone), transport protein steroidogenic acute regulatory protein (StAR), CYP11A1 (cholesterol side-chain cleavage), CYP17A1 (17α-hydroxylase-17,20-lyase), 3β-HSD (3β-hydroxysteroid dehydrogenase), 17β-HSD (17β-hydroxysteroid dehydrogenase), CYP19A1 (aromatase), CYP21 (21-hydroxylase), CYP11B1 (steroid 11β-hydroxylase), CYP11B2 (steroid 18-hydroxylase), 3α-HSD (3α-hydroxysteroid dehydrogenase), SULT (steroid sulfotransferase), STS (steroid sulfatase).

Steroidogenesis involves complex biosynthetic pathways converting cholesterol (**1**) into steroid hormones, including sex steroids (androgens, oestrogens, and progestogens) and corticosteroids (glucocorticoids and mineralocorticoids) ^29^. This process, which is finely tuned and controlled by the hypothalamus–pituitary–­steroidogenic glands axis, mainly occurs in the adrenal gland, gonads, and placenta. However, extra-glandular steroidogenesis is also known in local tissues such as the brain, immune cells, adipose tissue, skin, and thymus. Although it is still the object of debate within the scientific community, the physio-pathological role of extra-glandular steroidogenesis represents a clear indication of a highly integrated endocrine-nervous-immune circuit^27^^,^^28^^,^^30^. Steroidogenesis begins with the transport of the required precursor cholesterol (**1**) from the outer mitochondrial membrane to the inner membrane by the transport protein steroidogenic acute regulatory protein (STARD1) (Figure 1), which represents the rate-limiting step in the generation of steroid hormones^31^. In the inner mitochondrial membrane, the cytochrome P450scc enzyme (CYP11A1), also namely cholesterol side-chain cleavage enzyme or 20,22-desmolase, cleaves the cholesterol side chain to give pregnenolone (**2**, PGN), a step shared by both the classical and backdoor pathways^32^. In the classical pathway, pregnenolone undergoes several enzymatic transformations, primarily in the smooth endoplasmic reticulum of the adrenal cortex and gonads^33^^,^^34^. PGN (**2**) is converted, in the adrenal cortex and testes, to 17α-hydroxypregnenolone (17αOH-PGN, **3**) *via* 17α-hydroxylase activity of CYP17A1^35^. In the zona reticularis of the adrenal cortex gland, 17αOH-PGN (**3**) is then transformed into dehydroepiandrosterone (DHEA, **4**) through the action of the same enzyme CYP17A1, which now acts as a 17,20-lyase^36^. DHEA (**4**) is then converted to androstenedione (**5**) either directly, through oxidation by 3β-hydroxysteroid dehydrogenase (3β-HSD), or indirectly *via* androstenediol (**6**) ^37^. Androstenedione (**5**) is then reduced into testosterone (**7**, T) in peripheral tissues by 17β-hydroxysteroid dehydrogenase (17β-HSD) and, subsequently to dihydrotestosterone (DHT, **8**) by the enzyme 5α-reductase (or 3-oxo-5α-steroid 4-dehydrogenases) (Figure 1) ^38^. In tissues such as adipose, breast, and gonads, androstenedione (**5**) and T (**7**) can be converted to oestrone (**9**, E1) and oestradiol (**10**, E2), respectively, by the aromatase (CYP19A1) ^39^. Next, steroid sulfatase affords the corresponding C3-sulphate derivatives **11** and **12**, which represent the inactive and circulating metabolites of oestrogen hormones (Figure 1) ^40^. Furthermore, during pregnancy, E1 (**9**) and E2 (**10**) are substrates of 16α-hydroxylase and 15α-hydroxylase with the formation of oestriol (E3, **13**) and estetrol (E4, **14**) ^41^. PGN (**2**) can be also converted to progesterone (PG, **15**) and 17α-hydroxy-progesterone (17αOH-PG, **16**) by the action of 3β-HSD and CYP17A1. In the zona fasciculata of the adrenal gland, PG (**15**) and 17αOH-PG (**16**) are substrates of 21-hydroxylase (CYP21A2) that produce deoxycorticosterone (**17**) and 11-deoxycortisol (**18**), from which the glucocorticoid hormones corticosterone (**19**) and cortisol (**20**) are obtained through the action of steroid 11β-hydroxylase (CYP11B1) (Figure 1) ^42^. In the zona glomerulosa, aldosterone synthase (steroid 18-hydroxylase, CYP11B2) produces the mineralcorticoid hormone aldosterone (**21**) from corticosterone (**19**) (Figure 1) ^43^.

The backdoor pathway provides an alternative route to androgen production, bypassing T (**7**) to produce directly the potent androgen DHT (**8**) *via* 5α-DHP (**22**) and androsterone (**23**) (Figure 1). This pathway is particularly important during foetal development but is also implicated in certain cancers, like castration-resistant prostate cancer^44^.

Understanding these pathways and their regulation is essential to design therapeutic strategies that effectively curb the growth of hormone-sensitive tumours^45^. Indeed, specific enzymes like CYP17A1 (for its 17α-hydroxylase and/or the C17,20-lyase activity), 5α-reductase, aromatase and steroid sulfatase represent valuable targets in the treatment of hormone-dependent cancers^11^^,^^12^^,^^46^.

### Aromatase inhibitors

**Figure 2.**
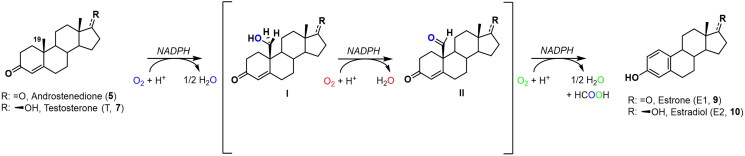
Catalytic mechanism of aromatase for the conversion of androgens **5** and **7** to oestrogens **9** and **10**.

Aromatase (CYP19A1, EC 1.14.14.14) catalyses the final and irreversible conversion of androstenedione (**5**) and T (**7**) into E1 (**9**) and E2 (**10**), respectively^47^. This transformation occurs mainly in the ovary, breast, and adipose tissue where CYP19A1 is largely expressed^48^. However, especially during menopause and *post*-menopause conditions, the conversion of androgens to oestrogens has been also observed in peripheral tissues such as the placenta, bone, skin, testis, and brain^49^. The catalytic cycle involves the sequential hydroxylation of the C19 methyl of androgens **5** and **7** in the presence of nicotinamide adenine dinucleotide phosphate hydrogen (NADPH) as co-factor and molecular oxygen as the final electron acceptor, through interaction with the haem iron (Figure 2) ^50^.

As well demonstrated, aromatase is over-expressed in breast cancer tissues and it is therefore considered an effective therapeutic target in oestrogen receptor-positive (ER+) breast cancers^51^. The development of the first selective aromatase inhibitors in the late '70 has represented one of the major successes in the fight against breast cancer^52^.

**Figure 3.**
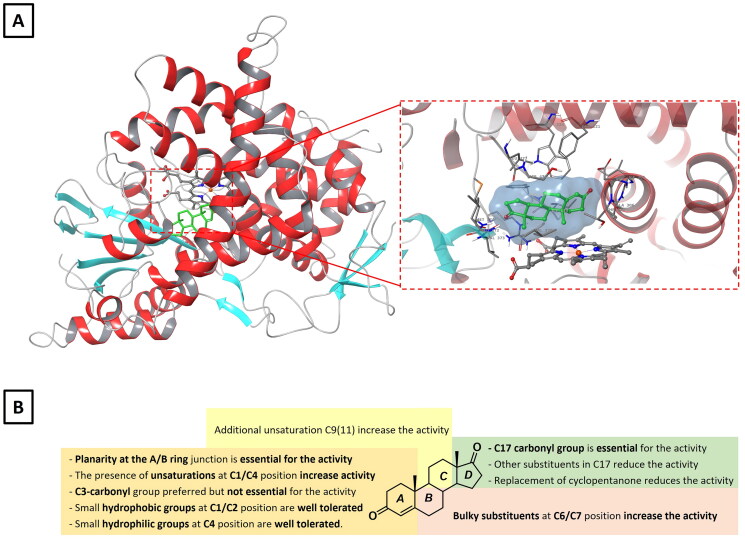
**A)** A ribbon diagram showing the structure of human placental aromatase (pdb: 3EQM) with a close-up view of the haem group and the bound androstenedione (**5**) at the active site. **B)** Structure–activity relationship (SAR) of steroidal aromatase inhibitors.

Aromatase inhibitors can be classified as steroidal and non-steroidal derivatives, and are further divided into generations, according to their chronological order of development^53–55^. Generally, steroidal inhibitors mime and compete with the endogenous substrate and are converted to highly reactive electrophilic intermediates (e.g. epoxide, oxirene and Michael acceptors) that covalently bind to the binding pocket of the enzyme, leading to irreversible inhibition. Interestingly, early structure-activity relationships (SAR) studies were carried out without the knowledge of the three-dimensional structure of the enzyme. Indeed, only in 2009 Ghosh *et al.* solved the crystal structure of the human aromatase (pdb: 3EQM), thus laying the molecular bases for the design of more potent and higher selective aromatase inhibitors^56^. Accordingly, several steroidal aromatase inhibitors have been discovered and developed over the years allowing drawing a precise SAR requirement for steroidal molecules (Figure 3B) ^53–55^^,^^57^. In particular, the planarity of the A/B ring and the presence of unsaturation(s) at the A and/or B rings are crucial for the inhibitory activity. On the other hand, the C3-carbonyl group does not seem to be critical for the activity while both small hydrophobic groups at the C1 position and small ­hydrophilic groups at the C4 position are well tolerated. Finally, bulky substituents at the C6- or C7-position fit within a hydrophobic cavity resulting in an increase of the inhibitory potency (Figure 3B) ^53–55^^,^^57^.

**Figure 4.**
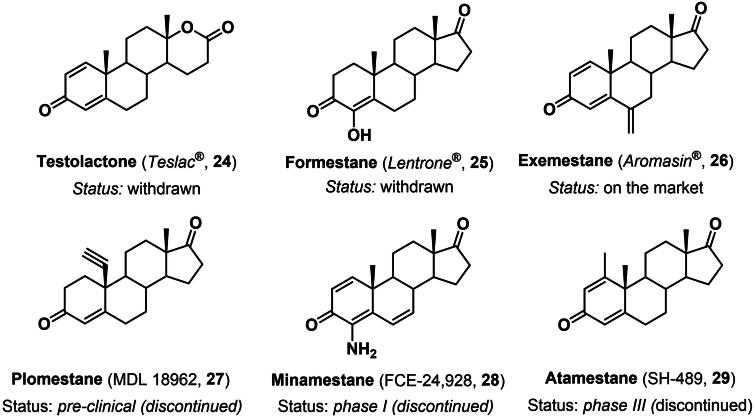
Steroidal aromatase inhibitors **24**–**26** approved for therapy and candidates **27**–**29** that reached clinical development stages.

The first steroidal aromatase inhibitor approved in therapy was testolactone (**24**), a synthetic analogue of T (**7**) where the usual five-membered D-ring of the steroidal scaffold was replaced by a δ-lactone moiety (Figure 4) ^58^. Testolactone (**24**) was approved by the FDA in 1970 and manufactured by Bristol-Myers Squibb under the brand name of Teslac^®^. The drug was an orally-active non-selective and non-competitive irreversible aromatase inhibitor able to reduce the circulating levels of oestrogen in *post*-menopausal women affected by breast cancer. Despite its low selectivity, testolactone (**24**) showed a good safety profile and was therefore used in therapy until 2008, when it was discontinued due to the emergence of newer and more selective inhibitors^58^. In the early 1990s, a second-generation analogue, namely formestane (**25**), was developed as the first steroidal selective aromatase inhibitor (Figure 4) ^59^. The compound was marketed under the brand name Lentrone^®^ by Ciba-Geigy Pharmaceuticals as an intramuscular injection, although the drug was never approved by the FDA and latterly withdrawn in Europe due to low potency, lack of specificity, and side effects^59^. In 1999, FDA approved exemestane (**26**) (Aromasin^®^), a 6-methylidene derivative of androstenedione developed at Farmitalia Carlo-Erba^60^ as the first orally-active, highly potent (IC_50_= 42 nM) and selective, non-competitive irreversible inhibitor (Figure 4). The drug was active at a low dosage (25 mg *per* day) and showed a good pharmacokinetic and safety profile, thus replacing tamoxifen as the golden standard therapy in the treatment of ER-positive breast cancers in *post*-menopausal women and advanced breast cancer unresponsive to tamoxifene^60^. Other analogues, such as plomestane (**27**), minamestane (**28**) and atamestane (**29**), entered clinical settings but never reached the market (Figure 4) ^61^.

**Figure 5.**
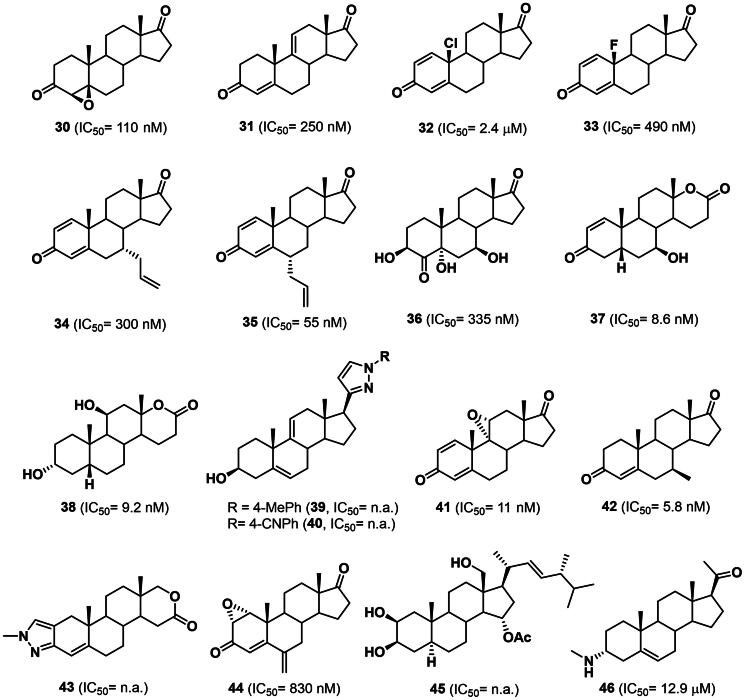
Structure and activity of steroidal aromatase inhibitors **30–46**. n.a.: not available

Despite limited oestrogenic side effects, the use of steroid aromatase inhibitors is sometimes associated with hot flashes, vaginal dryness, and headache^62^. In addition, drug resistance phenomena often occur during long-term therapy and treatment of recurrences^63^. Therefore, current efforts are directed towards the optimisation of the efficacy, selectivity and pharmacokinetic profile, as well as to extent their clinical application to other hormone-related pathologies, such as male infertility, bone depletion in elderly male and gynaecomastia^64^. Thus, in the last ten years, a number of steroids have been evaluated as anti-cancer aromatase inhibitors (Figure 5) ^53–55^.

In 2016, Varela and co-workers reported the synthesis of aromatase inhibitors by structural modifications of androstenedione (**5**), with derivatives **30** and **31** being the most promising compounds with IC_50_ of 110 and 250 nM, respectively (Figure 5) ^65^. Chlorination or fluorination at the C10-position of androst-1,4-dien-3,17-dione^66^, as well as alkylation at C6α− or C7α − position resulted in novel aromatase inhibitors **32**–**35** exhibiting IC_50_ values in the *sub*-micromolar range, with C6α-allyl analogue **35** being a nanomolar aromatase inhibitor (IC_50_= 55 nM) (Figure 5) ^67^^,^^68^. In 2021, two patent applications, one of which granted in 2024, reported the synthesis *via* whole-cells fungal biotransformation and the aromatase inhibitory activity of polyhydroxylated formestane and testolactone analogues^69^^,^^70^. Among them, derivatives **36**–**38** exhibited IC_50_ values in the *sub*-micromolar and low nanomolar range (Figure 5). In the same year, Banday *et al.* reported the preparation, the docking studies and biological appraisals of novel potential human aromatase inhibitors **39** and **40** characterised by the pregnenolone scaffold and a pyrazole ring at C17β-position (Figure 5) ^71^. Highly potent nanomolar aromatase inhibitors **41** and **42** (IC_50_= 11 nM and 5.8 nM, respectively) have been recently reported by Roleira and co-workers by epoxidation of androst-1,4-dien-3,17-dione at C9α,C11α-position or by insertion of a methyl group at C7β position of androstenedione (**5**) (Figure 5) ^72^. In 2023, Šestić *et al*. described the synthesis and biological evaluation on novel A- and D-ring modified androstane derivatives^73^. Among the synthesised derivatives, the pyrazole analogue **43** produced a significant red-shift of Soret peak in the absorption spectrum of recombinant human aromatase, thus suggesting a potential interaction with the haem cofactor. The same year, oxymestane-D1 (**44**), an analogue of exemestane (**26**) bearing an epoxide ring at C1α,C2α-position, was evaluated as multi-target anti-cancer drug (Figure 5) ^74^. Indeed, beyond the aromatase inhibitory activity in the nanomolar range, **44** resulted a potent ER antagonist and AR agonist. The compound was also able to promote the activity of caspases-7, 8, and 9, to reduce DNA synthesis in a thymidine incorporation assay and to decrease cell viability on a MTT assay by blocking cell cycle on the G0/G1 phase in oestrogen-positive cancer cell line MCF7 and breast cancer cell line resistant LTED.

An alternative strategy to design potent aromatase inhibitors is based on natural oestrogens and phytosterols (Figure 5). In this regard, in 2020 Abdelhameed and co-workers discussed the isolation and biological evaluation of a new ergosterol derivative from seagrass *Thalassodendron ciliatum*^75^. In particular, starting from the crude extract of *T. ciliatum*, thalassosterol (**45**) was isolated and evaluated in docking studies for its ability to bind the aromatase enzyme. The good superimposition of D-ring with the key residue Met374 of the binding site is responsible for the enzyme inhibition and would justify the cytotoxicity in different cell lines observed by SRB assay at micromolar concentration. The same year, various phytochemicals were isolated from *Sarcococca saligna* and tested for their inhibitory activity against aromatase. Among them, the steroidal alkaloid **46** showed an IC_50_ of 12.9 µM and a favourable binding pose as demonstrated by docking studies (Figure 5) ^76^.

Despite the significant efforts, none of these compounds has entered clinical investigations so far. Indeed, the use of aromatase inhibitors in therapy is still associated with a number of phenomena that needs to be further addressed. These include: a) the primary and secondary types of resistance, b) the ineffectiveness of aromatase inhibitors towards exogenous oestrogens, oestrogenic-like pollutants, phytoestrogens and endogenous androgens that act as partial ER agonists (e.g. androstenediol), and c) the inherent oestrogen sensitivity of some mutant ER+-tumours^77^. Based on this evidence, the search for effective anti-cancer drugs is being directed towards innovative multi-targeting mechanisms (*see* paragraphs 2.2 Steroid Sulfatase Inhibitors and 2.4 17α-Hydroxylase/17α,20-lyase Inhibitors).

### Steroid sulfatase inhibitors

**Figure 6.**
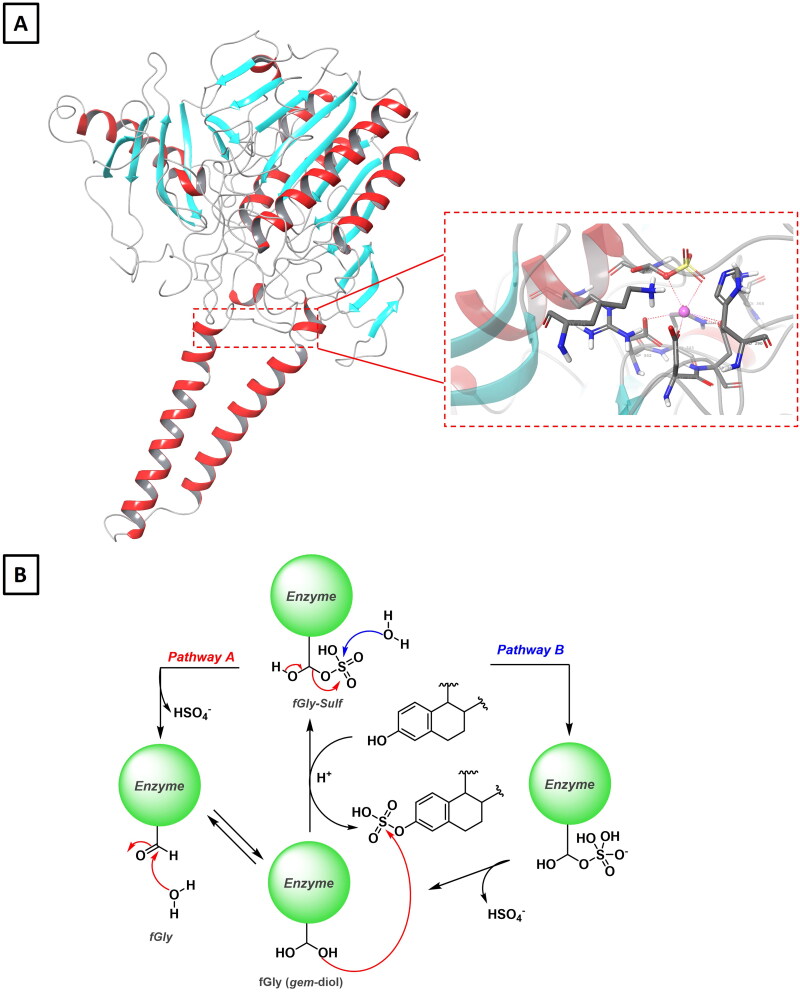
**A)** A ribbon diagram showing the structure of human placental steroid sulfatase (pdb: 1P49) with a close-up view of the catalytic site where a sulphated formylglycine (FGly75) residue in its *gem*-diol form coordinates a calcium ion. **B)** Addition-hydrolysis (*pathway A*) and transesterification–elimination (*pathway B*) mechanisms proposed for the hydrolysis of steroid sulphates.

Sulfatases are phase II metabolic enzymes that catalyse the hydrolysis of sulphate ester using 3-phosphoadenosine 5′-phosphate (PAP) as the acceptor of sulphate group^78^. Among the 17 sulfatases in the human genome^79^, steroid sulfatase (STS, EC 3.1.6.2), also known as arylsulfatase C (ARSC), is the enzyme responsible for the cleavage of sulphated steroids, including E1 (**9**), E2 (**10**), DHEA (**4**), PGN (**2**) and cholesterol (**1**) sulphates (Figure 1) ^80^. Highly water-soluble steroid sulphates, produced by the action of sulfotransferases, represent the main circulating steroidal metabolites that are almost inactive at their respective receptors. Therefore, the STS activity is crucial for determining the final level and the hormonal receptor activity, of pregnanes, estranes and androstanes in specific tissues^81^. STS is a transmembrane protein mainly associated with the endoplasmic reticulum and highly expressed in the placenta, adrenal glands, ovary, testis, bone, prostate, skin, and brain. (Figure 6A) ^82^^,^^83^. When a natural substrate binds the active site of the enzyme, a conformational change occurs thus shifting the hydrophobic helices close to the steroidal core. Upon binding of steroid sulphates, two putative catalytic mechanisms have been proposed. The addition-hydrolysis hypothesis (pathway A) starts with the decomposition of formylglycine (fGly) sulphate, thus releasing fGly which then reacts with a water molecule furnishing the hydrated formylglycine form (Figure 6B). On the other hand, in the transesterification–elimination mechanism (pathway B), the activation of FGly75 by a molecule of water occurs first, followed by the nucleophilic attack of the sulphur atom facilitated by a calcium ion. Finally, the release of the hydrolysed product and HSO_4_^-^ regenerates FGly75 (Figure 6B) ^82^.

STS desulfation of DHEA (**4**) and E1 (**9**) sulphates represents the main route for the formation of active oestrogens and androgens. Therefore, it is not surprising that several experimental evidences have clearly pointed out the therapeutic utility of STS inhibitors in the treatment of hormone-dependant cancers^84–87^. Indeed, high and/or abnormal levels of STS activity have been observed in almost all breast, prostate and endometrial cancers^88–90^. More recent studies have shown that STS is also present in colon carcinomas and, therefore, the reduction of circulating oestrogen in *post*-menopausal women is associated with a reduction in the risk of colorectal cancer^91^. Interestingly, STS is also found in the epidermis where it plays a crucial role in the local production of androgens. Therefore, STS inhibitors can be used for the local treatment of androgen-dependant inflammatory skin disorders as hirsutism, alopecia, acne and psoriasis^92^. Finally, the presence of STS in the central nervous system and its role in balancing sulphated and non-sulphated neurosteroids, which modulate γ-aminobutyric acid A (GABA-A) and *N*-methyl-D-aspartate (NMDA) receptors, may further expand the clinical applications of STS inhibitors for treating attention deficit hyperactivity disorder^93^^,^^94^.

**Figure 7.**
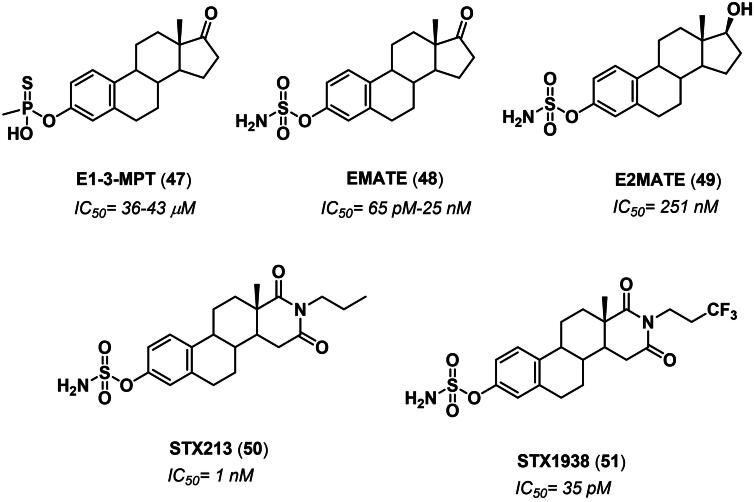
Structure of steroidal sulfatase inhibitors **47**–**51** that reached *pre*-clinical and clinical development.

Although the first reports on STS inhibition date back to the 1970s, the development of STS inhibitors has witnessed a remarkable acceleration in the mid-90s thanks to the pioneering works of Michael Reed and Barry Potter^87^. The design of this first generation of STS inhibitors was focussed on the bioisosteric replacement of the sulphate group of E1 sulphate, the endogenous substrate of the enzyme, with metabolically stable phosphonates, sulphonates, sulphonyl halides, sulphonamide and methylenesulfonyl groups^95^^,^^96^. As a result, these compounds act as potent and selective irreversible competitive STS inhibitors *via* sulfamoyl moiety transfer to the catalytic fGly residue^97^. Among the first-generation inhibitors based on this strategy, E1-3-MTP (**47**) ^98^, EMATE (**48**) ^96^, and E2MATE (**49**)^99^ represent the most successful derivatives, with E2MATE (**49**) that reached phase II clinical trial for the treatment of pain symptoms of endometriosis (Figure 7) ^100^. Second- and third-generation steroidal STS inhibitors, such as STX213 (**50**) and STX1938 (**51**), were developed in the early 2000s and *pre*-clinically evaluated in a mouse model of hormone-dependant breast cancer (Figure 7) ^101^^,^^102^.

**Figure 8.**
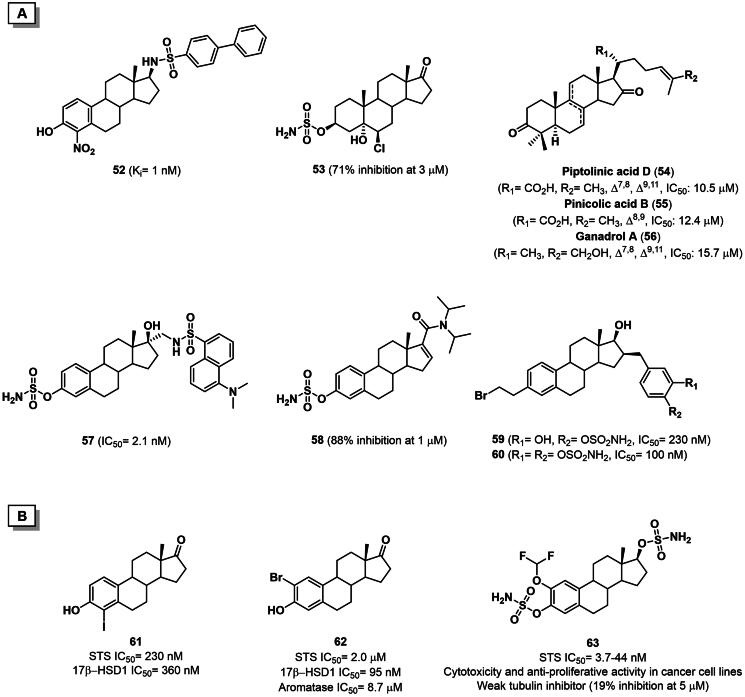
**A)** Structure and activity of steroidal sulfatase inhibitors **52–60**. **B)** Structure and activity of multi-target inhibitors **61–63**.

Since then, several A- and D-ring modified E1/E2 analogues from both synthetic and natural-occurring sources have been reported, thus contributing to define an extensive SAR analysis^97^^,^^103^. In 2015, a set of 17-arylsulfonamides of E2 was synthesised and evaluated as STS inhibitors. Among them, 4-nitro derivative **52** exhibited a K_i_ of 1 nM and *in vitro* antiproliferative activity in the NCI 60 cell line (Figure 8A) ^104^. In 2018, a 5α-hydroxy-6β-chloro derivative of androstane-3β-*O*-sulfamate **53** was found to exert 71% inhibition in the JEC-3 cell line when tested at 3 µM concentration^105^. Lanostane derivatives, namely piptolinic acid D (**54**), pinicolic acid B (**55**), and ganadrol A (**56**), were identified as micromolar STS inhibitors from a pharmacophore-based virtual screening campaign conduced on natural triterpenes used in Chinese folk medicine (Figure 8A) ^106^.

In 2020, Maltais and collaborators identified by QSAR studied two fluorescent dansyl-labelled E2 analogues with nanomolar STS inhibition activity (Figure 8A). Interestingly, compound **57** (IC_50_= 2.1 nM) was employed as a chemical probe in confocal microscopy to demonstrate its localisation in the endoplasmic reticulum where STS is mainly expressed^107^. The same year, the University of California patented a series of steroidal sulfamate derivatives and, among the claimed compounds, derivative **58** showed 88% STS inhibition when tested at 1 µM concentration on VCaP cell line^108^. The compound also exerted *in vitro* anti-proliferative activity on various breast cancer cell lines as well as *in vivo* growth inhibition in mouse models of prostate cancer (Figure 8A). In 2021, Poirier *et al.* identified compounds **59** and **60** as potent inhibitors (IC_50_ values of 230 nM and 100 nM, respectively) against STS from homogenised transfected HEK-293 cells (Figure 8A) ^109^.

So far, none of the STS inhibitors have reached the market, leaving room for the design of novel candidates. In this regard, a new trend in the field consists of the development of multi-target compounds capable of modulating different pathways or enzymes within the steroidogenic cascade (Figure 8B) ^97^. In 2018, Bacsa *et al.* reported the SAR study of 2- and/or 4-halogenated oestrone derivatives as multitarget steroidogenic enzyme inhibitors^110^. As a result, mono-halogenated derivatives were more active than their corresponding non-halogenated and di-halogenated analogues, with the iodine substituent exhibiting the best inhibitory activity. On the other hand, substitution at the C4 position gave good inhibition towards both STS and 17β-HSD1. Among the investigated compounds, derivative **61** displayed a dual action on STS and 17β-HSD1, with IC_50_ values in the high nanomolar range. In contrast, its corresponding 2-bromo analogue **62** was found to be a weak aromatase/STS/17β-HSD1 inhibitor, with IC_50_ values of 8.7, 2.0, and 0.095 μM, respectively (Figure 8B) ^110^. More recently, Potter and co-workers discussed the antiproliferative activity of a series of 2-difluoromethoxy-substituted sulfamate derivatives^111^. Derivative **63** was efficacious in both JEG-3 human choriocarcinoma lysate and whole-cell assays with IC_50_ values of 44 nM and 3.7 nM, respectively. Moreover, a synergistic cytotoxicity was observed in MCF-7 and MDA-MB-231 breast cancer cell lines (IC_50_ = 280 and 740 nM, respectively), along with the antiproliferative activity against six cancer cell lines. Finally, the compound exhibited weak inhibition of the colchicine binding to tubulin (19% inhibition at 5 μM) (Figure 8B) ^111^.

### 5α-reductase inhibitors

**Figure 9.**
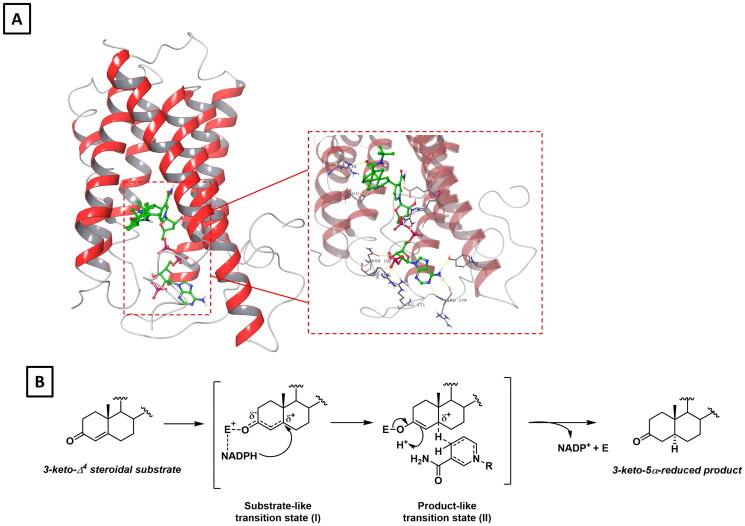
**A)** Crystal structure of human steroid 5-alpha-reductase 2 in complex with finasteride (pdb: 7BW1) with a close-up view of its binding at the active site. **B)** Proposed mechanism for 5α-reductase showing the 'substrate-like' (I) and 'product-like'(II) transition states.

Steroid 5α-reductases or 3-oxo-5α-steroid-4-dehydrogenases (S5αRs; E.C.1.3.99.5) are a family of membrane-bound enzymes the catalyse the hydrogenation at the ring A of endogenous Δ^4^–3-keto-steroids, including PG (**15**), deoxycorticosterone (**17**), corticosterone (**19**), aldosterone (**21**), androstenedione (**5**) and, most importantly, T (**7**) (Figure 1) ^112^. S5aR family comprises three isozymes, namely S5aR type 1–3. The three isoenzymes differ for tissue distribution, chromosomal location, biochemical and pharmacological properties, as well as substrate specificity and optimum operative pH values^113^. In particular, SRD5 type 1 is mainly expressed in sebaceous glands, epidermal keratinocytes, neurons of both the central and peripheral nervous system, adrenal glands and the prostatic epithelial cells. The enzyme operates at an optimum pH range of 6.0–8.5 and exerts less affinity for T (**7**) and PG (**15**) compared to isoform 2. On the other hand, SRD5 type 2 isozyme is mainly expressed in tissues and organs of the male urogenital tract. The type 2 isoform operates at pH range 5.0–5.5 and is responsible for the synthesis of about two thirds of circulating DHT (**8**) (Figure 9A) ^113^. Finally, SRD5 type 3, also known as polyprenol reductase, seems to play a minor role in steroidogenesis, being unable to reduce PG (**15**), androstenedione (**5**) and corticosterone (**19**), but still able to reduce T (**7**) to some extents. Interestingly, recent evidences suggest that the overexpression of S5aR type 3 isozyme is observed in breast cancer, lung adenocarcinoma, testicular seminoma, and castration-recurrent prostate cancer^114^. From a biochemical point of view, the activity of S5AR increases the susceptibility of the carbonyl group at the C3 position to be reduced and conjugated with hydrophilic moieties, facilitating their excretion^113^. On the physio-pathological stand-point, this irreversible transformation is pivotal for converting T (**7**) to the more potent DHT (**8**). Therefore, the use of S5αRs inhibitors is considered a valuable therapeutic approach to treat DHT-dependent diseases, including benign prostatic hyperplasia (BPH) and its progression to prostate cancer, as well as androgenic alopecia and acne^115^. However, due to their substrate promiscuity, the modulation of S5αRs activity is not merely limited to the endocrine regulation. Indeed, in the liver the S5αR-mediated conversion of endogenous corticosterone (**19**) to 5α-dihydrocorticosterone is important in glucose homeostasis^116^, while in the eye the same metabolite is involved in the production of aqueous humour^117^. Furthermore, S5αRs are involved in the production of neuroactive steroids (e.g. allopregnanolone) that modulate the behaviour and the reaction to environmental stress by activating the GABA-A receptor in the brain^118^.

The mechanism of S5AR requires NADPH as the co-factor and involves the interaction between the electrophilic residues of the active site of the enzyme with the 4-en-3-one moiety of the substrate (Figure 9B) ^119–121^. The ternary complex enzyme-NADPH-substrate thus formed activates the enone system, thereby generating a delocalised, transient carbocation at the C-5 position. Then, a hydride transfer from NADPH to the C-5 position of the steroidal scaffold occurs to the α-face of the delocalised carbocation, leading to the formation of an enolate intermediate. Upon protonation of the enolate at the β-face and subsequent solvolysis, the product is released along with the binary complex NADP^+^-enzyme. Finally, NADP^+^ is regenerated in its reduced form and the free enzyme is available for a new catalytic cycle (Figure 9B). Formally, two different transition states can be identified in this process: a 'substrate-like' transition state, in which the C-5 is sp^2^ hybridised, and a 'product-like' transition state, in which the C-5 already possesses anan sp^3^ hybridisation. These transition states can be therefore exploited for the design of specific inhibitor able to mime such interactions (Figure 9B) ^119^.

**Figure 10.**
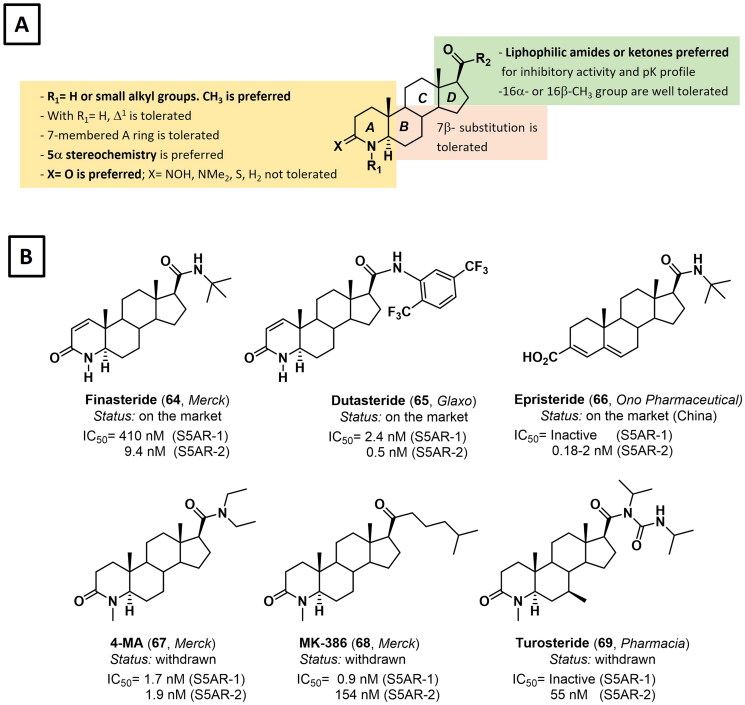
**A)** Structure–activity relationship (SAR) analysis of 4-azasteroids as inhibitor of steroidal 5α-reductases. **B)** Structure of 4-azasteroids **64**–**66** that reached the market and the clinical candidates **67**–**69**.

The search for effective inhibitors of 5α-reductase started in the early 80s at Merck with the development of several azasteroids obtained by structural modifications of the natural substrate T (**7**) ^122^. Initially, inhibitors were designed to mime both the DHT 'enol-like' (4-azasteroids) and the 'substrate-like' transition state (6- and 10-azasteroids). 4-Azasteroids are by far the most studied class of S5AR inhibitors, with three analogues on the market and several derivatives that reached clinical stages (Figure 10) ^119^^,^^123–126^. The tremendous efforts made by researchers in the early '90s led to define a robust SAR that finely tunes both potency and selectivity, as well as the pharmacokinetic properties (Figure 10A) ^119^^,^^123–126^. In 1992 these efforts culminated with the FDA approval of finasteride (Proscar^TM^, **64**) for the treatment of BPH. Finasteride (**64**) was patented by Merck in 1984 as a potent and type 2-selective competitive inhibitor of S5AR^127^. The compound has then obtained a second and a third approval by FDA, as oral low-dose monotherapy for the treatment of androgenetic alopecia (Propecia^®^), and in combination with tadalafil (Entadfi^®^) for the treatment of BPH (Figure 10B) ^128^^,^^129^. In 2001, dutasteride (**65**, Avodart^®^) was approved by the FDA as monotherapy or in combination with tamsulosin for the treatment of BPH^130^. Dutasteride (**65**) was patented in 1996 by Glaxo as a second generation of 4-azasteroid being two orders of magnitude more potent than finasteride (**64**) and almost equally active against type 1 and type 2 S5AR, with a partial inhibition activity against type 3 S5AR. This higher inhibitory activity results in nearly complete *in vivo* suppression of DHT production (up to 98%) when administered at 0.5 mg once *per* day, compared to 71% inhibition obtained by finasteride (**64**) given at 5 mg daily^131^.

More recently, dutasteride (**65**) has been also approved for the treatment of scalp hair loss in South Korea and Japan. Moreover, although still not approved by the FDA, it is currently used off-label for the treatment of androgenetic alopecia in men and hirsutism in *pre*-menopausal women^132^^,^^133^. Notably, dutasteride (**65**) is currently under clinical investigation for additional therapeutic indications, including prostate cancer and *pre*-menstrual dysphoric disorder (Figure 10B) ^134^^,^^135^. In 2000, epristeride (**66**, Aipuliete^®^) was approved in China for the treatment of enlarged prostate. However, despite its potent inhibition of 5α-reductase type II (IC_50_= 0.18–2 nM), the compound has poor ability to reduce *in vivo* the circulating levels of DHT, thus preventing its approval by EMA and FDA^136^. Other analogues based on the 4-azasteroid template that hashas been investigated in clinical stages without reaching the market include 4-MA (**67**), MK-386 (**68**) and turosteride (**69**) (Figure 10B) ^131^. 6- and 10-Azasteroids, though less studied than 4-azasteroids, have demonstrated promising enzymatic inhibition and potential therapeutic applications, particularly as anti-cancer and antimicrobial agents. However, their lower *in vivo* stability compared to 4-azasteroids reduces bioavailability and shortens their duration of action^137^^,^^138^.

**Figure 11.**
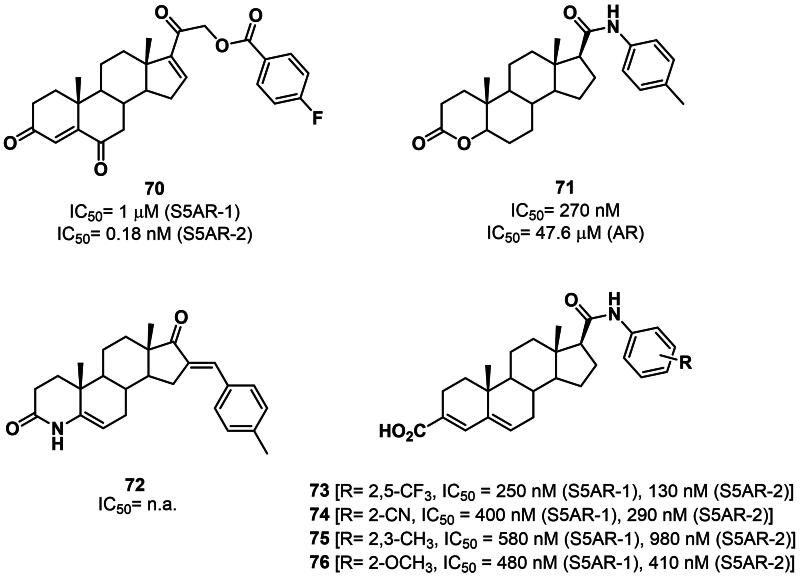
Structure and activity of steroidal 5α-reductase inhibitors **70**–**76**. n.a.: not available

Beyond azasteroids, other classes of steroidal inhibitors of S5ARs developed over the years include unsaturated or aromatic 3-carboxysteroids derivatives, D-ring lactam derivatives, D-ring lactone analogues of DHEA, C3- or C17β-modified progesterone and 16-dehydropregnenolone derivatives, and numerous natural products^119^^,^^123–126^^,^^131^. More recently, the design of novel derivatives has been driven by the necessity to mitigate side effects (e.g. sexual dysfunction, gynaecomastia, depression), particularly for therapeutic indications such as alopecia, hirsutism, and prostate cancer prevention, where a high risk-to-benefit ratio is unacceptable. Thus, in 2015, a set of *p*-fluorobenzoyloxy-21-esters of pregnenolone were synthesised and tested for their inhibitory activity against S5AR-1 and −2^139^. Derivative **70** was selected as the hit compound displaying *in vitro* inhibitory activity and selectivity against S5AR-2 in the low nanomolar range. The activity was also confirmed *in vivo*, as the compound significantly reduced the weight of the prostate and seminal vesicles when administered in combination with T (**7**) to castrated hamsters (Figure 11) ^139^. A scaffold-hopping approach has been recently exploited by Lao and co-workers to drive the design of novel 3-oxo-4-oxa-5α-androst-17β-amide derivatives^140^. Among the tested compounds, derivative **71** showed 4-fold higher cytotoxicity against LNCaP (androgen-dependent) than PC-3 (androgen-independent) prostate cancer cell line. Furthermore, compound **71** exhibited nanomolar inhibition of 5α-reductase and acted as an AR antagonist with a micromolar IC_50_ value. Interestingly, the compound showed a longer plasma half-life and better bioavailability compared to the benchmark drug finasteride (**64**) (Figure 11) ^140^. In 2018, Brito *et al.* reported the synthesis and characterisation of novel 16-arylidene-4-aza-androstene derivatives^141^. Among the investigated analogues, compound **72** exerted *in vitro* anti-proliferative activity with no cytotoxicity detected in healthy human fibroblasts and in the PC-3 cell line. Moreover, molecular docking studies evidenced a putative inhibition against 5α-reductase (Figure 11) ^141^.

More recently, Lao *et al*. reported the design and synthesis of novel androst-3,5-dien-3-carboxylic acids bearing aromatic amides at position C17^142^. Compounds **73–76** have been evaluated for their *in vitro* inhibitory activity against S5AR-I and II, as well as for their *in vivo* ability to reduce the weight of the prostate gland. From this study, compound **73** emerged as a potent dual inhibitor of S5ARs showing beneficial effects in a rat model of BPH. ADME studies also evidenced a favourable pharmacokinetic profile, thus making compound **73** a valuable lead compound for further *in vivo* appraisals (Figure 11) ^142^. It is worth noting that most of the 5α-reductase inhibitors developed so far were designed without the availability of the three-dimensional structure of the enzyme. In this regard, the recent release of the first crystal structure of human S5AR type II (PDB: 7BW1)^120^ is expected to drive the design of novel, more potent, and selective 5α-reductase inhibitors.

### 17α-hydroxylase/17α,20-lyase (CYP17) inhibitors

**Figure 12.**
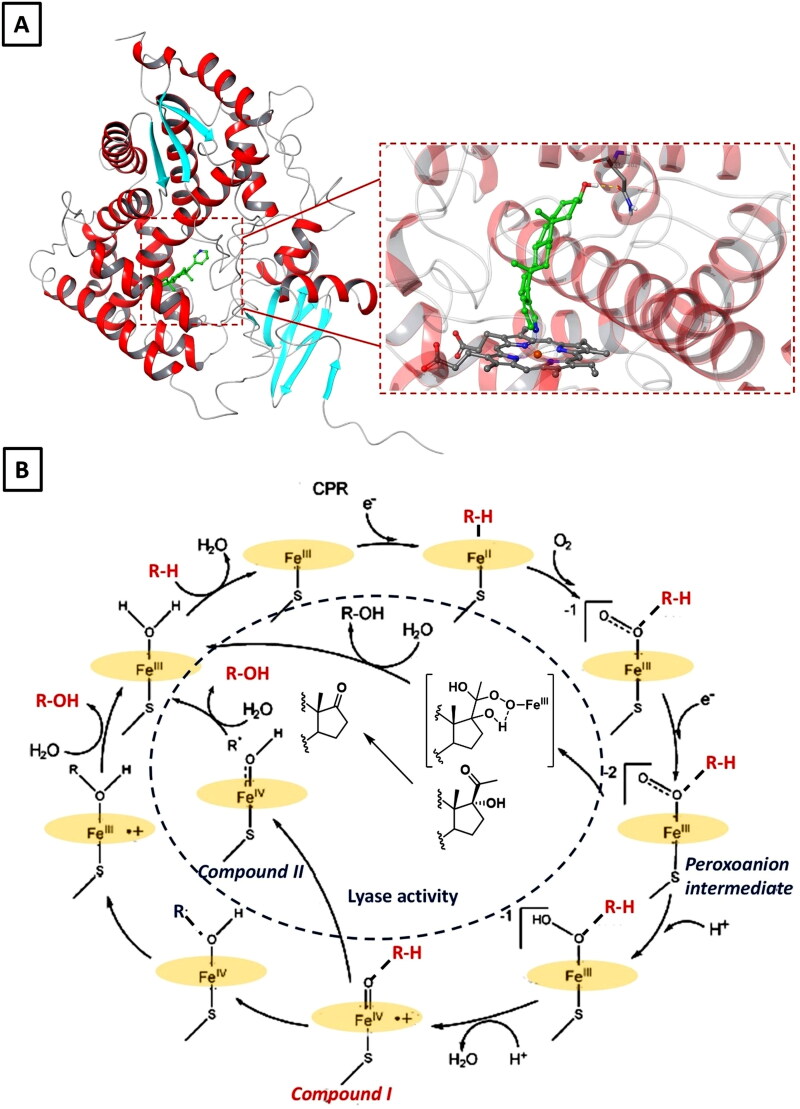
**A)** Crystal structure of human CYP17A1 in complex with abiraterone (pdb: 3RUK) with a close-up view of its binding at the active site. **B)** Proposed catalytic cycle of CYP17A1 for both 17α-hydroxylation and 17,20-lyase activities (in the blue dotted circle). The structures of compound I, compound II and the peroxoanion intermediate are shown.

CYP17A1 is a member of the cytochrome P450 superfamily and plays a pivotal role in the biosynthesis of both glucocorticoids and androgens^143^. Indeed, the enzyme catalyses two sequential reactions in both the gonads and adrenal glands: the 17α-hydroxylation (EC 1.14.14.19) of PGN (**2**) and PG (**15**) to form the corresponding 17α-hydroxy-pregnane intermediates **3** and **16**, and the 17α,20-lyase reaction (EC 1.14.14.32) that cleaves the C21 side chain of these intermediates into DHEA (**4**) and androstenedione (**5**), the precursors of androgen hormones T (**7**) and DHT (**8**) (Figure 1) ^144^. This bifunctional enzymatic activity, which was initially attributed to two separate enzymes, occurs within a single active site^145^. While the 17α-hydroxylation step contributes to corticosteroid synthesis, the 17,20-lyase activity is essential for androgen production. The inhibition of CYP17A1 results in the total blockage of androgen production, within both the classical and backdoor steroidogenic pathways, in tests, adrenal glands and prostate cells^146^. Therefore, the selective inhibition of lyase activity, without disrupting 17α-hydroxylation needed for corticosteroid synthesis, is a valuable therapeutic approach for developing drugs able to target androgen biosynthesis in hormone-driven malignancies as metastatic castration-resistant prostate cancer (CRPC) ^144^. In pursuit of this goal, the elucidation of CYP17A1 crystal structures has been instrumental in understanding its catalytic mechanism, thereby guiding researchers in the design of selective inhibitors.^35^ The first CYP17A1 crystal structure was obtained in 2012 and revealed the conserved P450 fold with 12 major α-helices labelled A-L (Figure 12A). CYP17A1 embeds into the endoplasmic reticulum *via* a transmembrane helix and a hydrophobic region on the distal side of the catalytic domain. Since then, structural studies of numerous crystal structures of CYP17A1 in complex with substrates and inhibitors have provided detailed insights into the catalytic mechanism and binding modes at the active site^145^. The catalytic cycle begins with the enzyme in its 'resting state', where the haem iron is in its ferric (Fe³^+^) state (Figure 12B) ^143^. Binding of the substrate PGN (**2**) or PG (**15**) displaces a water molecule positioning the substrate within the active site. An electron from NADPH, transferred *via* cytochrome P450 reductase, reduces the haem iron to the ferrous (Fe^2+^) state. Molecular oxygen then binds to the ferrous haem, forming a ferrous-dioxygen complex. A second electron from NADPH and subsequent protonation yield a peroxy intermediate, which is further protonated to form a highly reactive iron-oxo species, known as compound **I**. For 17α-hydroxylation, compound **I** abstract a hydrogen atom from the C17 position of the substrate, facilitating the hydroxylation and producing 17α-OH-PGN (**3**) or 17α-OH-PG (**16**).

For the lyase reaction, two different mechanisms have been proposed. In the first pathway, compound **I** abstracts hydrogen from the substrate forming compound **II** and a substrate radical (R^.^). On the other hand, the second mechanism involves the peroxoanion intermediate that attacks the substrate to form a peroxyhemiketal transition state, thus releasing the lyase product. The efficiency of the lyase reaction depends on specific cofactors, such as cytochrome b5, which enhances the reaction by stabilising the intermediates (Figure 12B) ^143^.

CYP17 inhibitors can be classified into steroidal and non-steroidal compounds based on their chemical structures^147^. In particular, steroidal inhibitors are often designed as analogues of the endogenous substrates, typically modified at the C17 position. These inhibitors are further divided into mechanism-based inhibitors and competitive inhibitors. Competitive inhibitors, which reversibly bind to the active site, are further categorised as type I or type II based on their interaction with the haem iron of the enzyme. Type I inhibitors bind to the enzyme by displacing the coordinated water molecule at the haem iron. This displacement induces a pentacoordinate state of the iron and results in a characteristic Soret band shift to approximately 390 nm. These inhibitors occupy the steroid-binding site, effectively competing with natural substrates. On the other hand, type II inhibitors coordinate directly with the haem iron through a heteroatom, such as nitrogen or oxygen, forming a coordinate bond that stabilises a hexacoordinate iron state and induces a Soret band shift to 421–430 nm^148^^,^^149^.

**Figure 13.**
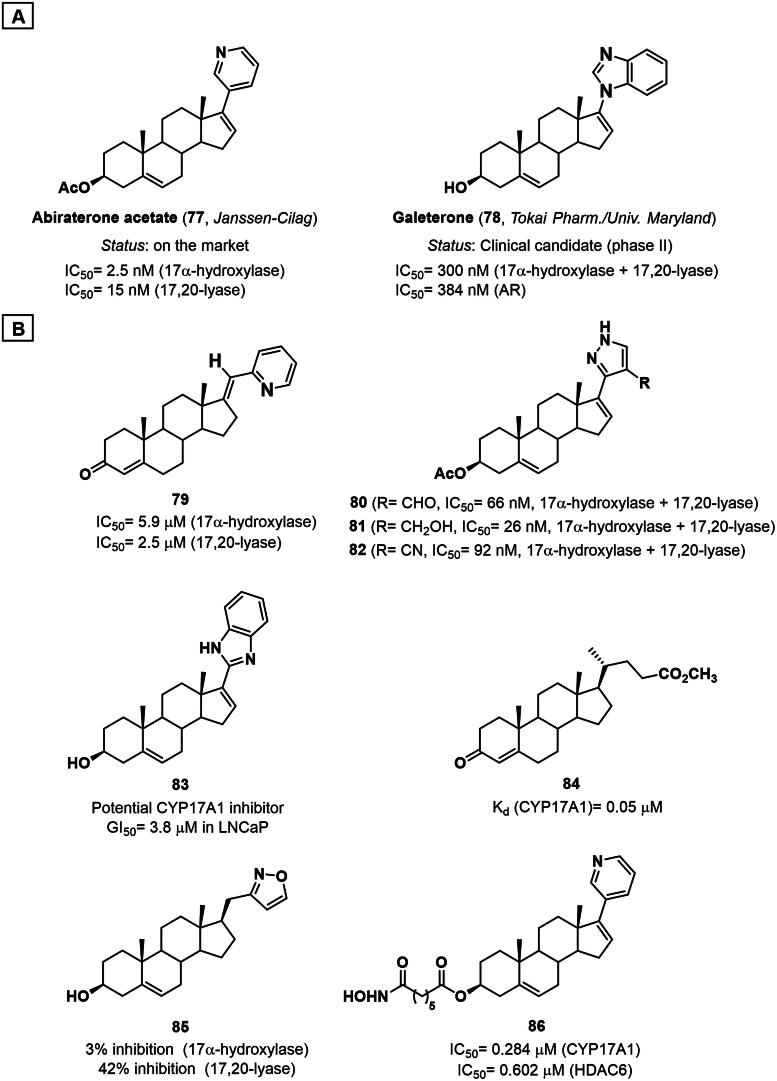
**A)** Structure of the approved steroidal CYP17A1 inhibitor abiraterone acetate (**77**) and the clinical candidate galeterone (**78**). **B**) Structure and activity of CYP17A1 steroidal inhibitors **79**–**86**.

A breakthrough in the field came with the development of abiraterone acetate (**77**, Zytiga^®^), a steroidal inhibitor derived from Δ^5,16^-pregnadienolone that incorporates a pyridyl group at the C17 position (Figure 13A). The acetate pro-drug form of abiraterone was approved by the FDA in 2011 for the treatment of CRPC after demonstrating efficacy in clinical trials^150^. The ability of abiraterone acetate (**77**) to suppress plasma levels of testosterone underscores its potency as an irreversible inhibitor of both 17α-hydroxylase (IC_50_=2.5 nM) and 17,20-lyase (IC_50_= 15 nM) activities^151^. In addition to its role as an inhibitor of CYP17A1, abiraterone acetate (**77**) also acts as an inhibitor of 17β-HSD, 3β-HSD, CYP11B1, CYP21A2, and other xenobiotic metabolising CYP450s^152^. These off-target effects along with the non-selective inhibition of 17α-hydroxylase activity are responsible for the numerous side effects associated with its therapeutic use. Moreover, not all patients respond to abiraterone and resistance may frequently occur through up-regulation of CYP17A1, induction of AR and AR splicing variants^153^^,^^154^. Therefore, these limitations pushed for the search for inhibitors with greater selectivity for lyase activity while sparing hydroxylase function. Beyond abiraterone acetate (**77**), structural optimisation of CYP17 inhibitors has focused on modifying the C17 substituent by incorporating various heterocyclic groups, such as thiazole, furan, thiophene, and imidazole, to enhance binding affinity and selectivity^155^^,^^156^. These heterocycles generally interact with the enzyme active site, leveraging their lone-pair electrons to form coordinate bonds with the haem iron. Among the clinically advanced compounds, galeterone (**78**) not only inhibits CYP17A1 but also acts as a selective androgen receptor degrader (SARD) (Figure 13A) ^157^. Although the candidate failed to meet its primary endpoints in a large-scale phase III study, it remains of scientific interest due to its multiple mechanisms of action with several back-up AR-degraders and down-regulators currently under investigation^158–160^.

During the last 10 years, several studies have contributed to further investigate the SAR of steroidal CYP17A1 inhibitors, shedding light on the molecular discriminants for selective inhibition of the lyase activity over the hydroxylase activity of the enzyme. In 2015, Szécsi and co-workers developed a radiosubstrate *in vitro* incubation method for the distinct determination of 17α-hydroxylase and C17,20-lyase activity on CYP17A1 deriving from rat testicular homogenate^161^. The authors tested a set of steroidal picolyl and picolinylidene derivatives and observed a stronger enzyme inhibition in the 17-picolinylidene series. In particular, 17-picolinyliden-androst-4-en-3-one derivative **79** was the most effective inhibitor with activity on both the 17α-hydroxylase and C17,20-lyase (Figure 13B). These data were rationalised by considering that the trigonal geometry of the double bond at the C17-C20 position in the 17-picolinylidene series extends the π-electron conjugation of the heterocyclic aromatic ring, thus promoting its binding to the haem iron and therefore enhancing the inhibitor activity^161^. In 2016, Kovàcs *et al.* reported the preparation, antiproliferative and CYP17A1 inhibitory activity of a series of 17–(4′-formyl)pyrazolylandrosta-5,16-dienes (Figure 13B) ^162^. The synthesised compounds were tested *in vitro* on four human adherent breast cancer cell lines, revealing that seven out of ten compounds exhibited >50% growth inhibition at 10 µM (IC_50_= 1–6 µM). Moreover, compounds **80–82** also showed selective inhibition of rat testicular C17,20-lyase, with IC_50_ values ranging from 26 to 92 nM (Figure 13B) ^162^. Recently, seven novel analogues of abiraterone bearing oxazoline, benzoxazole and benzimidazole moiety at C17 position have been reported^163^. Docking studies using the active site of human CYP17A1 revealed that all the synthesised compounds were able to form stable complexes with the enzyme. The steroid skeleton is superimposed on the abiraterone pose, while the nitrogen atom coordinates with the haem iron. Among the reported analogues, derivative **83** emerged as a valuable multi-target candidate, being also able to destabilise the helix 12 of the AF2 domain of the AR receptor, similarly to galaterone (**78**), and exert a potent anti-proliferative effect in prostate carcinoma LNCaP and PC-3 cells (Figure 13B) ^163^.

An interesting study by Dzichenka and collaborators revealed for the first time that bile acid derivatives can decrease the rate of hydroxylation or completely inhibit the CYP17A1 activity, exhibiting micromolar or *sub*-micromolar affinity^164^. SAR analysis based on *in vitro* and *silico* data showed that the affinity of bile acid derivatives for the enzyme is related to the degree of hydrophobicity. In this regard, planar Δ^4^ bile acids with a ketone group at the C3 position and a C24 side chain bearing an ester group (e.g. compound **84**) showed the highest affinity for the enzyme, while the presence of more polar side chain or substituents at C7 and C12 positions confers higher K_d_ values (Figure 13B) ^164^.

More recently, 3β-hydroxy-Δ^5^-steroids bearing substituted isoxazole fragments at their side-chain have been proposed as potential anti-prostate cancer agents^165^. In particular, derivative **85** was identified as the most promising compound able to selectively promote the inhibition of 17,20-lyase activity with only a minimal inhibitory effect on 17α-hydroxylase (Figure 13B). Molecular docking studies confirmed that compound **85** was characterised by a binding pose and energy similar to abiraterone (**76**), with the isoxazole ring oriented towards the iron ion of the haem group similarly to the pyridine moiety of abiraterone (**76**). Moreover, based on the structural similarity of the novel derivatives with galeterone (**78**), AR-transcriptional activity was evaluated at concentrations ranging from 2 to 50 μM in both antagonistic and agonistic modes using a luciferase assay with an AR-dependent reporter cell line (ARE14). As a result, compound **85** showed a moderate dose-dependent AR-antagonistic activity (50% at 50 µM), although the molecule did not reach the downstream AR signalling levels exhibited by galeterone (35% at 10 μM) (Figure 13B) ^165^. In 2024, Sharma *et al.* explored the dual inhibition of CYP17A1 and histone deacetylase-6 (HDAC6) as a promising, novel strategy to identify molecules with therapeutic potential for the treatment of glioblastoma (Figure 13B) ^166^. A series of abiraterone-installed hydroxamic acids were designed, prepared and tested with compound **86** being identified as a low micromolar dual inhibitor (IC_50_ of 0.284 µM and 0.602 µM against CYP17A1 and HDAC6, respectively). The compound demonstrated a strong anti-glioblastoma activity, particularly in temozolomide-resistant cells, by inducing apoptosis, oxidative stress, and DNA damage response. Molecular modelling was instrumental to rationalise its efficacy, while *in vivo* studies confirmed a significant tumour suppression activity with minimal toxicity (Figure 13B) ^166^.

## Conclusions and future outlooks

The therapeutic potential of steroidal enzyme inhibitors in oncology has been well-established through their ability to target key enzymes in steroidogenesis, thereby modulating hormone levels that drive the progression of hormone-dependent cancers. In this review article, we have reported and discussed the research studies and development efforts directing our attention to the discovery, molecular basis, and therapeutic applications of steroidal enzyme inhibitors in hormone-dependent cancers. We have evidence that despite the progress made in the last ten years, challenges remain to be solved in mitigating drug resistance and managing off-target effects, which limit the long-term efficacy and patient outcomes. Addressing these limitations requires a multifaceted approach^19^. First, combinatorial therapies that pair steroidal enzyme inhibitors with agents targeting parallel or compensatory pathways offer a promising strategy to overcome adaptive resistance mechanisms. For example, the co-administration of anti-androgen therapies with agents inhibiting growth factor signalling or DNA repair pathways has shown a potential to delay resistance in advanced prostate cancer^167^^,^^168^. Similarly, in breast cancer, combining aromatase inhibitors with CDK4/6 inhibitors or PI3K/AKT pathway modulators may enhance therapeutic effectiveness^169^^,^^170^. Precision medicine approaches that use biomarkers to identify patients most likely to benefit from such combinations are being actively investigated^171^. Second, the development of multi-target agents represents a compelling avenue to enhance the pharmacological profile of steroidal inhibitors. By simultaneously targeting multiple enzymes or receptors involved in steroid metabolism or signalling, these agents can provide broader-spectrum activity, while potentially reducing the need for combination regimens^172^. Rational medicinal chemistry approaches, such as molecular hybridisation or scaffold hopping optimisation, as well as omic analysis coupled with machine-learning approaches will be instrumental in achieving this goal^173–175^. Third, innovative targets and therapeutic approaches can help to mitigate both adverse effects and resistance phenomena^19^. In this regard, innovative selective androgen receptor down-regulators/degraders (SARDs) ^176^, and androgen- and oestrogen receptor-targeted proteolysis targeting chimaera (antiAR- and antiER-PROTAC) are currently under development^177^^,^^178^. Other novel potential therapeutic targets in hormone-dependent cancers, which are not yet clinically validated but can be modulated by steroidal compounds, include enzymes such as NAPE-PLD^179^ and carbonic anhydrases^180^, protein-protein interactions like the EpH-ephrin system^181^, and orphan nuclear receptors/transcription factors^182^. By integrating these innovative strategies, future research could usher in a new era of personalised and precision-based hormone cancer therapy, with steroidal inhibitors likely playing a pivotal role in improving patient outcomes.

In conclusion, the continuing evolution of steroidal enzyme inhibitors as anticancer agents will depend on synergic efforts aimed at addressing resistance and side effects through combinatorial therapies, multi-target strategies, and cutting-edge drug design approaches. With sustained innovation, these agents hold the promise to deliver transformative benefits for patients battling hormone-dependent cancers.

## Data sharing is not applicable – no new data is generated

Data sharing is not applicable to this article as no new data were created or analysed in this study.
